# Integrated Analysis of miRNAs Associated With Sugarcane Responses to Low-Potassium Stress

**DOI:** 10.3389/fpls.2021.750805

**Published:** 2022-01-04

**Authors:** Nannan Zhang, Xiaomin Feng, Qiaoying Zeng, Huanzhang Lin, Zilin Wu, Xiaoning Gao, Yonghong Huang, Jiayun Wu, Yongwen Qi

**Affiliations:** ^1^Guangdong Sugarcane Genetic Improvement Engineering Center, Institute of Nanfan & Seed Industry, Guangdong Academy of Sciences, Guangzhou, China; ^2^College of Agriculture and Biology, Zhongkai University of Agriculture and Engineering, Guangzhou, China

**Keywords:** sugarcane, microRNA, low-potassium stress, target genes, high-throughput sequencing

## Abstract

Sugarcane is among the most important global crops and a key bioenergy source. Sugarcane production is restricted by limited levels of available soil potassium (K^+^). The ability of plants to respond to stressors can be regulated by a range of microRNAs (miRNAs). However, there have been few studies regarding the roles of miRNAs in the regulation of sugarcane responses to K^+^-deficiency. To understand how these non-coding RNAs may influence sugarcane responses to low-K^+^ stress, we conducted expression profiling of miRNAs in sugarcane roots under low-K^+^ conditions *via* high-throughput sequencing. This approach led to the identification of 324 and 42 known and novel miRNAs, respectively, of which 36 were found to be differentially expressed miRNAs (DEMs) under low-K^+^ conditions. These results also suggested that miR156-x/z and miR171-x are involved in these responses as potential regulators of lateral root formation and the ethylene signaling pathway, respectively. A total of 705 putative targets of these DEMs were further identified through bioinformatics predictions and degradome analyses, and GO and KEGG enrichment analyses revealed these target mRNAs to be enriched for catalytic activity, binding functions, metabolic processes, plant hormone signal transduction, and mitogen-activated protein kinase (MAPK) signaling. In summary, these data provide an overview of the roles of miRNAs in the regulation of sugarcane response to low-K^+^ conditions.

## Introduction

Potassium is a key nutrient essential for the growth and development of plants (Maathuis, 2009), with potassium ions (K^+^) being present at high levels within cells wherein they regulate osmotic pressure and control the activation of key enzymes including those associated with photosynthesis (Clarkson and Hanson, 1980), protein synthesis, stomatal closure (Chérel and Gaillard, 2019), osmoregulation, phloem transport (Patrick et al., 2001), and a range of other processes (Leigh and Wyn Jones, 1984). K^+^ is crucial for sucrose loading into the phloem (Deeken et al., 2002), and K^+^-deficient sugarcane plants exhibit significantly impaired photosynthate exposure from leaves relative to that observed in K^+^-sufficient plants (Hermans et al., 2006; Hawkesford, 2012). K^+^ has also been highlighted as a key determinant of grapevine (*Vitis vinifera*) growth, yields, and resultant wine quality (Mpelasoka et al., 2003). K^+^ also regulates the ability of plants to tolerate a range of abiotic stressors directly and indirectly (Zörb et al., 2014; Du et al., 2019), including drought, salinity (Anschütz et al., 2014; Shabala and Pottosin, 2014), and many others (Cakmak, 2005; Li et al., 2011). High levels of K^+^ are conducive to appropriate osmotic adjustments under low soil water potential conditions by means of increasing cellular osmolyte concentration (Grzebisz et al., 2013) and limiting stomatal water loss (Peiter, 2011).

The roots are the primary source of K^+^ uptake in plants (Liu et al., 2020), but only a small proportion of the potassium present within the soil is in the form of available K^+^, and as such, K^+^ deficiency has become a major global threat to sustainable agricultural planting efforts (Römheld and Kirkby, 2010; Wang and Wu, 2015; Ye Z. et al., 2020). It is therefore vital that the genetics and regulation of plant low-K^+^ stress responses be studied in detail to facilitate the development of more K^+^-efficient crop cultivars.

MicroRNAs (miRNAs) are small RNA molecules (20–24 nt) that lack coding potential and that are expressed across plant species wherein they can facilitate the post-transcriptional downregulation of target genes. In so doing, miRNAs can function as important regulators of diverse physiological processes including abiotic stressor responses (Jeong and Green, 2013; Zhang, 2015). In *Arabidopsis*, for example, miR393 is robustly upregulated under cold, drought, NaCl, and ABA treatment conditions, whereas these same four treatments suppressed miR389a1 expression (Sunkar and Zhu, 2004). There is also evidence that miR292 is upregulated under dehydration conditions in a range of plants including *Medicago truncatula* (Wang et al., 2011), common bean (*Phaseolus vulgaris*) (Arenas-Huertero et al., 2009), and rice (*Oryza sativa*) (Zhao et al., 2007). Many miRNAs exhibit stressor-specific expression profiles, such as miR319, which is induced in response to cold stress but is unaffected by exposure to NaCl or ABA (Sunkar and Zhu, 2004).

Certain miRNAs have been shown to be responsive to nutrient deprivation (Paul et al., 2015), modulating N-deficiency (Liang et al., 2012; Sinha et al., 2015), phosphate (Pi)-deficiency (Kuo and Chiou, 2011; Du et al., 2018; Bao et al., 2019), magnesium-deficiency (Liang et al., 2017), and K^+^-deficiency responses (Zeng et al., 2019; Ye Z. et al., 2020). When Pi levels are low, for example, miR399 undergoes upregulation mediated by the MYB family transcription factor PHR1, whereupon the miR399 target gene *PHO2* is downregulated, facilitating enhanced Pi uptake and translocation in *Arabidopsis* (Fujii et al., 2005; Aung et al., 2006). Argonaute1 (AGO1) is an RNA splicing mediator that is suppressed by miR-168 (Qi et al., 2005; Xian et al., 2014). When exposed to low-K^+^ stress, tomato (*Solanum lycopersicum*) plants exhibit the upregulation of SlmiR-168a and corresponding AGO1 downregulation that alters miRNA responses and bolsters K^+^ deficiency tolerance (Liu et al., 2020).

Sugarcane (*Saccharum officinarum* L.) is an economically important crop that is harvested for consumption and as a major source of sugar and bioenergy (Nonato et al., 2001). Available K^+^ levels, however, can severely limit sugar production (Zeng et al., 2015). The advent of novel sequencing technologies has led to an increasingly detailed understanding of plant molecular biology (Varshney et al., 2009; Edwards and Batley, 2010), and such plant transcriptomic sequencing offers an invaluable opportunity to understand the molecular basis for gene expression patterns in specific agricultural contexts (Wang et al., 2009). Prior transcriptomic analyses of K^+^-deficient sugarcane plants have been conducted (Zeng et al., 2015; Feng et al., 2020), but how miRNAs regulate responses to K^+^ deficiency remains unclear. Conducting analyses of the specific miRNA-mediated regulatory mechanisms associated with low-K^+^ stress may provide valuable opportunities to advance the sugarcane industry. To that end, we herein chose Yuetang 55 (YT 55, also named as YT 99-66), a low-K^+^ tolerance sugarcane cultivar (Huang et al., 2013), and conducted a small RNA sequencing analysis of sugarcane roots following low-K^+^ exposure, after which candidate miRNA regulators of low-K^+^ responses were identified, and functional analyses of the targets of these miRNAs were conducted to better understand how sugarcane plants respond to K^+^ deficiency.

## Materials and Methods

### Plant Materials and Treatment

The Yuetang 55 (YT55) sugarcane cultivar used in these analyses was obtained from the Guangdong sugarcane Genetic Improvement Engineering Center, Institute of Bioengineering, Guangdong Academy of Sciences (Guangzhou, China), and the new plant variety (Yueshentang 2009001) was provided by the Department of Agriculture and Rural Affairs of Guangdong Province. All YT55 plants were cut into single bud setts that were sterilized using 5% carbendazim and germinated in quartz. Hydroponic culture techniques were then used to grow seedlings at 30°C under natural light in a greenhouse. Seedlings were cultured using modified Magnavaca’s solution (Zeng et al., 2015), which was replaced weekly. Following a 2-week period, all plants that had 8–10 adventitious roots were transferred to a low-K^+^ nutrient solution containing 0.1 mM KCl. Root samples from three replicate plants were then isolated after 0, 6, 12, 24, 48, and 72 h, and were snap-frozen with liquid nitrogen prior to storage at –80°C.

### Small RNA Sequencing

Trizol (Invitrogen, CA, United States) was used to extract total RNA from triplicate root samples, after which an RNA Nano 6000 Assay Kit and an Agilent Bioanalyzer 2100 instrument (Agilent Technologies, CA, United States) were used based on provided directions to quantify RNA levels. Small RNAs (18–30 nt) were then enriched *via* polyacrylamide gel electrophoresis (PAGE) and collected. Following 3′ and 5′ end adaptor ligation, small RNAs were reverse transcribed *via* PCR amplification, and those PCR products between 140 and 160 bp in size were enriched to yield a cDNA library. After quality assessment, these libraries were sequenced using an Illumina HiSeq 2000 instrument.

### Data Processing and Analysis

Raw RNA-seq data were cleaned prior to downstream analysis by removing reads containing low-quality bases, 5′-adapters and poly-A tails, and reads <18 nt long or that lacked a 3′ adapter and small RNA sequence fragments. Cleaned read data have been deposited into the National Center for Biotechnology Information (NCBI) Sequence Read Archive (SRA) database (accession number: PRJNA687913).

Cleaned reads were aligned to small RNAs in the GeneBank database (Release 209.0^1^), Rfam database (Release 14.3^2^), and the reference transcriptome (a novel YT55 PacBio transcriptome, accession number: PRJNA688942). After this, we removed sequences corresponding to snoRNAs (including rRNA, scRNA, snoRNA, snRNA, and tRNA), as well as those that may have been fragments generated *via* mRNA degradation or that mapped to repeated sequences. Known miRNAs were those that were found to be conserved across species, while the miRDeep2 software was used to identify novel miRNA candidates based upon their genomic positions and hairpin structures.

### Differentially Expressed microRNA Identification

All miRNA expression levels were assessed in the format of transcripts per million (TPM) using the formula: TPM = Actual miRNA counts/Total counts of clean tags × 10^6^. Those miRNAs that exhibited similar patterns of expression were clustered with the Short Time-series Expression Miner (STEM) software, with a *P* < 0.05 as the threshold for significance.

Differentially expressed miRNAs were identified with the edgeR package^3^ based upon normalized read counts in different samples. Those miRNAs with a fold change (*FC*) ≥ 2 and *P*-value < 0.05 were considered to be DEMs.

### Target mRNA Prediction and Functional Analysis

In this study, the target genes of identified miRNAs were predicted *via* both *in silico* and degradome sequencing, using the single-molecule long-read transcriptome of YT55 as a reference transcriptome. Target prediction was performed using the Patmatch software (Version 1.2^4^). To verify these potential target mRNAs, one degradome library was constructed from T24 roots of YT55 plants, and the sequencing and miRNA target analyses were carried out as per methods detailed by Ye Y. et al. (2020). The YT55 Pacbio transcriptome was used as a reference for miRNA target cleavage site validation. The cleaned degradome sequencing data have been deposited into the NCBI SRA database together with the miRNA transcriptome sequencing data (accession number: PRJNA687913). Functional categorization and miRNA target gene pathway enrichment analyses were conducted using the GO and KEGG databases in order to identify key metabolic and signal transduction pathways associated with these differentially expressed genes (DEGs).

### qPCR-Based Validation of microRNAs

RNAiso Plus (TaKaRa) was used to isolate total RNA from root samples based on provided instructions, after which qPCR and stem-loop qPCR were used to validate miRNA and target gene expression patterns using approaches previously detailed by Varkonyi-Gasic et al. (2007) and Zhang N. et al. (2018). Primers used for this analysis are compiled in Supplementary Table 13. An ABI 7500 Fast Real-Time PCR instrument was used for all analyses, with β-tubulin (Zeng et al., 2015) serving as normalization controls for relative expression analyses. The 2^–ΔΔCT^ approach was used to quantify relative gene expression levels.

### Accession Numbers

Clean sugarcane YT55 miRNA transcriptome and degradome sequencing data, as well as PacBio full-length transcriptome sequencing data from the present study are available in the NCBI Sequence Read Archive with the accession numbers PRJNA687913 and PRJNA688942, respectively.

## Results

### Small RNA Sequencing of Sugarcane Roots

We began by conducting the high-throughput sequencing of miRNAs identified in sugarcane roots exposed to low-K^+^ levels (0.1 mM K^+^) for 0, 6, 12, 24, 48, and 72 h (termed samples CK, T6, T12, T24, T48, and T72, respectively). A total of three biological replicates were prepared per sample condition, and these six respective samples yielded average raw read counts of 19,167,487, 24,564,586, 23,304,763, 17,754,932, 16,822,809, and 16,808,881, respectively. Following the removal of low-quality reads and those that were under 18 nt or over 30 nt in length, average clean read numbers obtained from these samples were 12,773,238, 11,484,108, 11,170,478, 11,474,947, 13,020,623, and 12,289,477, respectively. Statistical data pertaining to these 18 libraries are compiled in Supplementary Table 1.

Clean reads were aligned to small RNAs included in the Rfam and Genebank databases using the Bowtie tools, after which all ribosomal RNA (rRNA), transfer RNA (tRNA), small nuclear RNA (snRNA), and small nucleolar RNA (snoRNA) were removed, as were other non-coding RNA (ncRNA). For further details regarding the miRNA identification process, see Supplementary Table 1.

### Known and Novel microRNA Identification and Analyses

**FIGURE 1:**
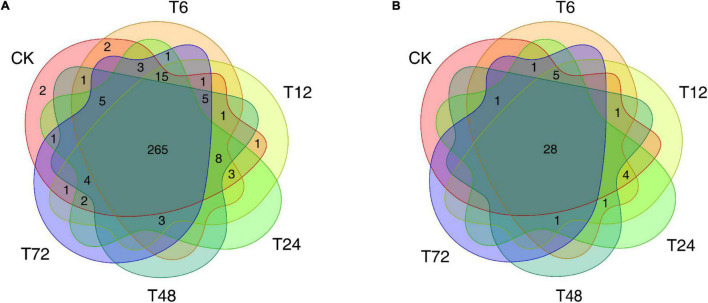
The distribution of miRNAs in six sugarcane root samples. **(A)** The distribution of the known miRNAs. **(B)** The distribution of the novel miRNAs. CK, T6, T12, T24, T48, and T72 correspond to 0, 6, 12, 24, 48, and 72 h after low-K^+^ treatment, respectively.

The identification of known and novel miRNAs within our sequencing data was conducted using the Yuetang55 (YT55) sugarcane cultivar PacBio full-length transcriptome dataset as a reference source, enabling us to map 20,883,828 total reads successfully. This led to the identification of 324 known miRNAs (Supplementary Table 2) following the mapping of these cleaned reads to the miRbase database, as well as 42 putative novel miRNAs which were predicted based on the architectural features corresponding to unannotated small RNA (sRNA) tags (Supplementary Table 3). Of the 324 known miRNAs, 265 were detected in all 18 root samples (Figure 1A and Supplementary Table 4), while 28 of the novel miRNAs were present across all libraries (Figure 1B and Supplementary Table 5).

**FIGURE 2:**
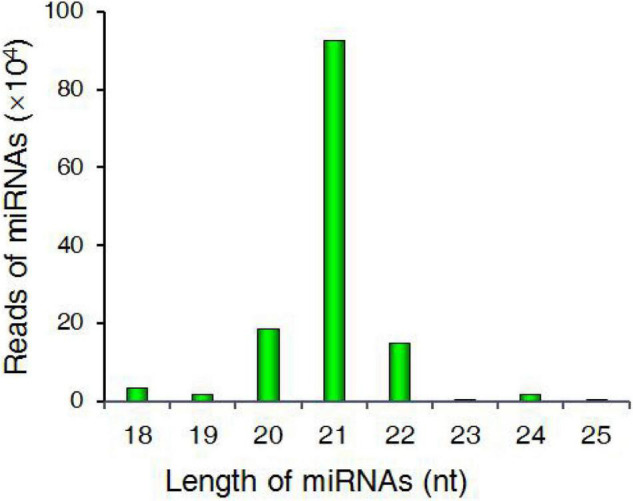
Statistics corresponding to identified miRNAs with different sizes in sugarcane roots.

Approximately 82% of these miRNAs were in the 21–24 nt size range, with 21 nt being the most common length (69.69%) (Figure 2). The most abundant nucleotide among the putative miRNAs identified herein was 5′-uridine (Supplementary Figure 1 and Supplementary Table 6).

### Identification of microRNAs That Were Differentially Expressed Under Low-Potassium Conditions

**FIGURE 3:**
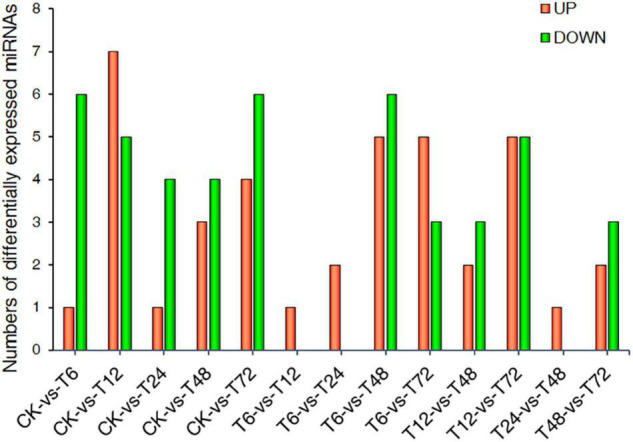
Statistics corresponding to miRNAs differentially expressed between two samples. CK, T6, T12, T24, T48, and T72 correspond to 0, 6, 12, 24, 48, and 72 h after low-K^+^ treatment, respectively.

Differentially expressed miRNAs (DEMs) that exhibited expression pattern changes upon low-K^+^ treatment were next identified. A total of 36 DEMs were defined *via* this approach (Figure 3 and Supplementary Table 7).

**FIGURE 4:**
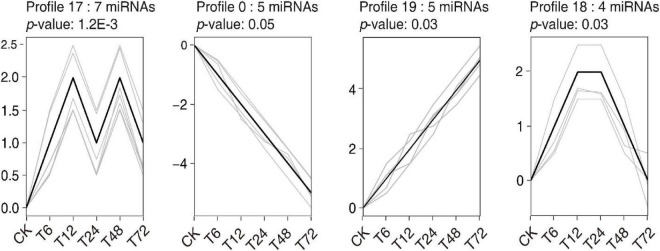
Significant expression trend profiles (with the cut-off criteria of *p* ≤ 0.05) for DEMs in sugarcane roots after low-K^+^ treatment. The x-axis represents time after low-K^+^ treatment, from 0 to 72 h. The y-axis corresponds to log2 fold changes in miRNA expression.

A Short Time-series Expression Miner (STEM) cluster analysis was performed to assess the temporal dynamics of miRNA expression following exposure to low-K^+^ conditions, resulting in all DEMs being grouped into 13 clusters (Figure 4, Supplementary Figure 1, Supplementary Table 8). Those miRNAs in clusters 17 and 19 were induced by low-K^+^ treatment, whereas those in clusters 0 and 2 were inhibited under these conditions. The miRNAs in cluster 19 exhibited time-dependent increases in expression under low-K^+^ conditions, whereas the opposite trend was observed for miRNAs in cluster 0. Other clusters did not exhibit consistent trends in expression patterns over time following exposure to low K^+^ levels. As such, we infer that miRNAs in different clusters play distinct roles in regulating sugarcane root responses to K^+^ deficiency.

### Identification and Functional Enrichment Analysis of Differentially Expressed miRNA Putative Target Genes

To better understand the functional importance of these DEMs, we predicted and verified their corresponding target genes based upon the YT55 reference transcriptomic dataset. A total of 18,314,716 raw reads representing 7,825,545 unique raw reads were generated from the degradome sample. After removing the reads lacking adaptors and short reads (<15 nt after removing 3′ adaptors), 7,787,991 unique reads (99.52% of all unique reads) were successfully mapped to 19,621 unigenes (96.71% of all 20,288 reference transcripts). The 6,573 miRNA cleavage sites were represented as target plots (T-plots) corresponding to 51, 35, 2,078, 800, and 3,609 miRNA-target pairs in categories 0, 1, 2, 3, and 4, respectively (Supplementary Table 9), including 705 genes targeted by 36 DEMs (Supplementary Table 10). Among the target genes of DEMs, PB.7532.1 and PB.7964.1, two targets of miR1848-z, were identified as putative voltage-gated potassium channel subunit beta genes likely to be directly involved in the absorption and transport of K^+^.

**FIGURE 5:**
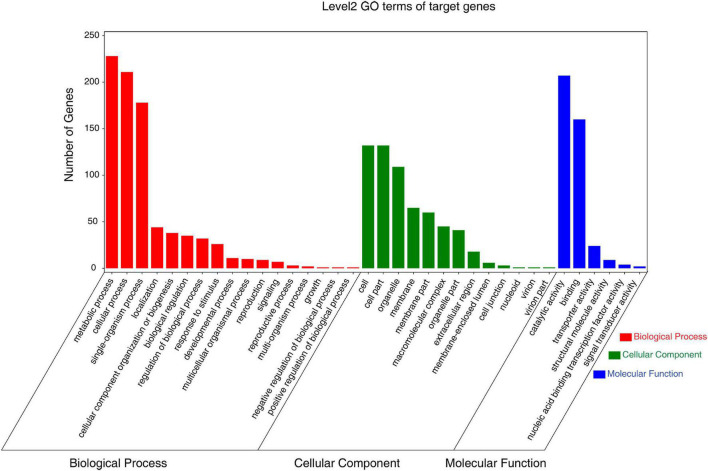
Gene ontology (GO) analysis of the predicted targets of differentially expressed miRNAs and most abundant biological process (red), cellular component (green), and molecular function (blue) GO terms.

GO and KEGG enrichment analyses of all DEMs were then used to classify the roles of these putative target genes under low-K^+^ conditions. GO analyses revealed these DEM targets to be associated with diverse biological processes, cellular components, and molecular functions (Figure 5 and Supplementary Table 11). The majority of these targets were associated with metabolic, cellular, and single-organism biological processes, binding functions, catalytic activity, and with cell and organelle compartments.

**FIGURE 6:**
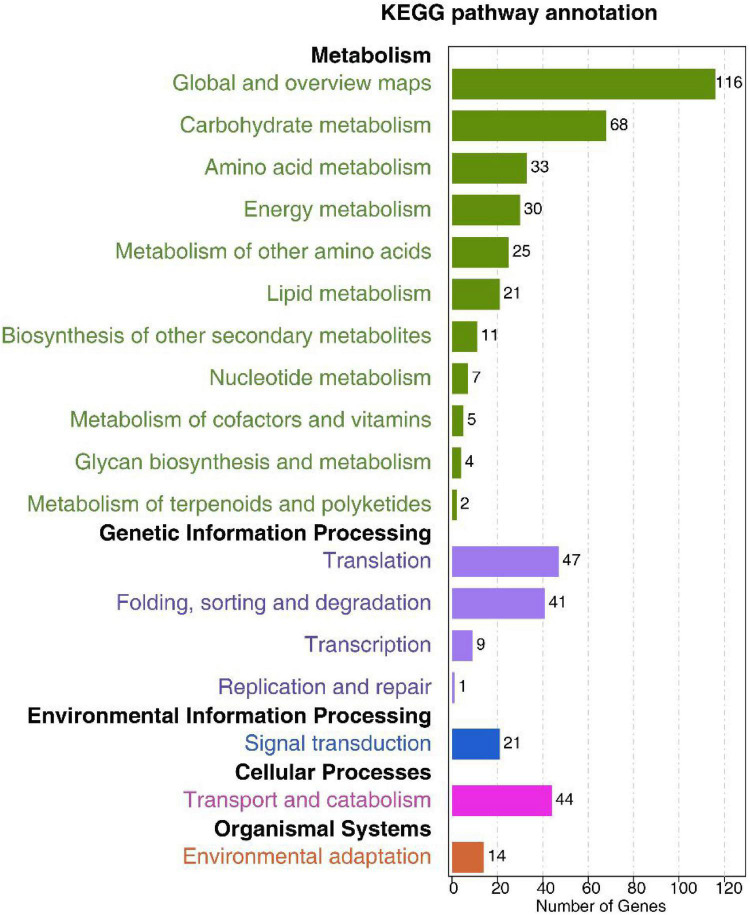
Low-K^+^ treatment-responsive differentially expressed miRNAs in terpenoid biosynthesis-related KEGG pathways.

KEGG pathway analyses revealed these predicted DEM targets to be associated with 15 significantly enriched pathways (*p* < 0.05). The majority of these targets were associated with metabolism, genetic information processing, environmental information processing, and cellular processes. Targets associated with metabolism pathways were enriched in the global and overview maps, carbohydrate metabolism, and amino acid metabolism. Moreover, lipid metabolism was also enriched (Figure 6 and Supplementary Table 12). The plant hormone and mitogen-activated protein kinase (MAPK) signal transduction pathways involved in plant responses to environmental changes were also significantly enriched for these target genes (Supplementary Table 12).

### Validation of Differentially Expressed miRNAs Associated With Sugarcane Responses to Low-K^+^ Conditions

**FIGURE 7:**
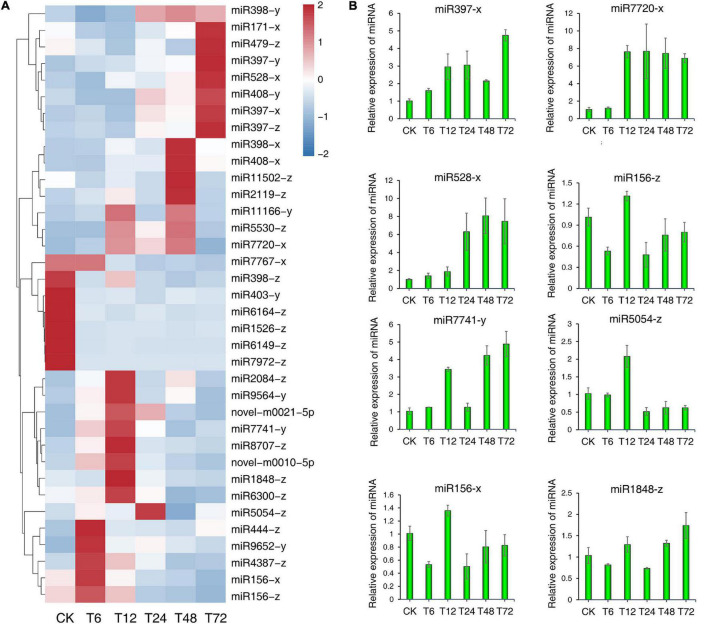
Validation of 8 differentially expressed miRNAs (DEMs) in sugarcane roots under low-K^+^ treatment by stem-loop RT-qPCR. **(A)** Heatmap of the relative expression levels of all 36 DEMs. **(B)** Relative expression levels of 8 DEMs detected by stem-loop RT-qPCR.

We observed the significant differential expression of 36 DEMs in roots in response to low-K^+^ treatment over time relative to control samples (S0, Figure 7A). Regulatory targets for these DEMswere identified based upon the abundance of mature miRNAs. To validate our small RNA sequencing results, we performed stem-loop qPCR to evaluate the expression patterns of 8 of these 36 DEMs with high count rates (Figure 7B), revealing results largely consistent with those from our sequencing analyses. For example, miR397-x exhibited peak expression levels at 72 h following low-K^+^ treatment, with a secondary peak at 12 h. Similarly, miR528 was expressed at the lowest levels during the early stage of treatment and maximally expressed at 72 h. However, there were some differences between stem-loop RT-qPCR and sequencing results. In accordance with the sequencing results, an expression peak corresponding to miR7741-y and miR1848-z was evident at 12 h after exposure to low-K^+^ conditions, although a second expression peak corresponding to these two DEMs was observed at 72 h *via* stem-loop RT-qPCR. These differential miRNA expression patterns may suggest that they play distinct time-dependent roles in controlling sugarcane root low-K^+^ stress responses.

## Discussion

Levels of K^+^ are one of the primary determinants of plant growth, development, and stress responses such that they are generally maintained at a stable concentration within plant cells (White, 2013; Demidchik, 2014; Zörb et al., 2014; Shabala, 2017). Soil deficient in available K^+^, however, has become an increasingly serious threat to global agricultural production efforts (Römheld and Kirkby, 2010; Wang and Wu, 2015; Liu et al., 2020). In plants, low-K^+^ stress was reported to impair photosynthesis (Trankner et al., 2018), growth (Hafsi et al., 2017), and tolerance to saline conditions (Hafsi et al., 2017). K^+^ deficiency can also increase JA concentrations (Cao et al., 2006; Armengaud et al., 2010), in addition to altering hormone signaling pathways including the gibberellin (GA), auxin, and abscisic acid (ABA) pathways (Hetherington, 2018). These changes likely coincide with altered miRNA expression. Prior research efforts have clarified those miRNAs that govern responses to low-K^+^ stress in tomatoes (Liu et al., 2020), foxtail millet (Cao et al., 2019), barley (Zeng et al., 2019; Ye Z. et al., 2020), and wheat (Thornburg et al., 2020). Sugarcane is not only a sweet tropical fruit but also a critical crop for sugar and bioenergy production efforts, in addition to being a “K^+^-favoring” crop such that low K^+^ levels will impair sugar accumulation, crop yield, and stress responses in affected plants (Nonato et al., 2001). Previous studies have clarified the physiological and biochemical processes (Zeng et al., 2019), comparative transcriptomic changes (Zeng et al., 2015), and high-affinity K^+^ transporters (*HAKs*) (Feng et al., 2020) associated with sugarcane responses to low-K^+^ stress. How miRNAs influence these responses, however, remains to be clarified, and transcriptomic analyses of changes in sugarcane miRNA expression profiles under low-K^+^ stress conditions may aid in the breeding of sugarcane cultivars able to tolerate K^+^ deficiency.

To detect miRNAs involved in sugarcane responses to K^+^ deficiency, we collected sugarcane root samples at a range of time points and conducted a transcriptomic analysis of the miRNAs identified therein. A length analysis revealed the majority of miRNAs in these samples to be 21 nt long, followed by 20 and 24 nt. In *Arabidopsis* (Jatan et al., 2020), hot pepper (*Capsicum annuum*) (Hwang et al., 2013), and trifoliate orange (*Citrus trifoliata*) (Song et al., 2010), most miRNAs have been reported to be 24 nt in length, whereas in sugarcane these miRNAs have been reported to be 21 nt (Ferreira et al., 2012) or 24 nt long (Li et al., 2017). Overall, the abundance of miRNAs of different lengths was attributed to differences in underlying physiological conditions.

Modern sugarcane cultivars are interspecific hybrids, with only 10–15% of their chromosomal content being derived from *S. spontaneum* (Zhang J. et al., 2018). As such, rather than utilizing the *S. spontaneum* genome as a reference source for miRNA identification, we instead utilized PacBio full-length transcriptomic data, leading to the detection of 324 and 43 known and novel miRNAs in our sugarcane root samples, respectively. Of these, 36 miRNAs were found to be differentially expressed as a function of time following low-K^+^ treatment (Figure 2). More DEMs were downregulated than upregulated at all time points following low-K^+^ exposure other than 6 h. To better understand the temporal dynamics of such DEM expression, a time-series analysis that grouped these miRNAs into 13 clusters was performed (Figure 4, Supplementary Figure 2, Supplementary Table 8). The miRNAs in clusters 0 and 19 were opposites of one another with respect to their expression patterns, as were those in clusters 1 and 18. This suggests that these miRNAs may play divergent roles in regulating low-K^+^ stress responses. A target analysis revealed that the targets of miRNAs in cluster 19 may be involved in plant responses to oxidative stress, including targets such as PB.10073.1 (Fujibe et al., 2006), PB.2586.1 (Overmyer et al., 2000), and PB.11689.1 (identified as peroxidase 5), which were identified as respective targets of miR408-y, miR528-x, and miR397-z. In addition, several targets of miRNAs in cluster 0 were associated with plant hormone pathways, including PB.5129.1 (Wang et al., 2000; Uraji et al., 2012) and PB.797.2 (Feng et al., 2003), both of which were targets of miR156-z (Figure 4 and Supplementary Tables 8, 10).

Comparative analyses of DEM expression patterns revealed some differences between sequencing and stem-loop RT-qPCR results. Certain DEMs including miR397-x, miR7720-x, miR528-x, miR156-z/x, and miR5054-z exhibited similar expression patterns in these two datasets but with expression peaks appearing at different points. However, the relative expression of miR7741-y and miR1848-z in the T48 and T72 samples analyzed by stem-loop RT-qPCR differed from that observed in the heatmap. This difference may be due to the differences in the algorithms employed by these two methods (Zou et al., 2020).

From a functional perspective, miRNAs can suppress target gene expression *via* RNA-induced silencing complex (RISC)-mediated translational repression and mRNA degradation (Bartel, 2004; He and Hannon, 2004; Jatan et al., 2020). In total, 705 putative targets of these 36 DEMs were identified. GO analyses suggested that these DEMs were involved in a range of sugarcane responses to low-K^+^ stress, and these results were consistent with differentially expressed genes (DEGs) previously reported by Zeng et al. (2015), with the exception of the “single-organism process” GO term being significantly enriched specifically in the present study. Identified genes associated with metabolic processes may also critically regulate low-K^+^ stress responses. Interestingly, targets of miR5054-z (PB.750.2, PB.58.1, PB.1452.1, PB.6875.1, and PB.1935.1), novel-m0021-5p (PB.13335.2), and miR7767-x (PB.4032.1) were identified as aquaporins, tonoplast intrinsic proteins (TIPs), and plasma membrane intrinsic proteins (PIPs), which are involved in water transport and the utilization of various physiological substrates (Weig et al., 1997; Ma et al., 2004; Groszmann et al., 2017; Supplementary Tables 8, 10). Aquaporins are also likely involved in plant responses to low-K^+^ stress.

Herein, we utilized the KEGG database to map the biological pathways associated with these target genes. Most of the target genes were enriched in amino acid, carbohydrate, and lipid metabolism pathways, highlighting the importance of these major nutrients in plant responses to low-K^+^ stress. The results also revealed 21 total targets associated with signal transduction pathways. Of these, 14 were associated with the MAPK signaling pathway and 10 were associated with plant hormone signaling (Supplementary Table 12). Plant hormones are essential regulators of plant responses to a range of stressors. Under low-K^+^ conditions, signaling pathways associated with phytohormones, reactive oxygen species (ROS) production, and Ca^2+^ can be activated (Kim et al., 2010; Wang et al., 2018). Ethylene, for example, has previously been shown to induce ROS generation and to thereby control plant low-K^+^ stress responses by modulating early signal transduction activity (Kim et al., 2010). Therefore, we speculated that the changes in the ROS, Ca^2+^, MAPK, and phytohormone signaling pathways in sugarcane plants under low-K^+^ stress can result in changes in DEM expression. The MAPK pathway signaling is conserved in both plants and animals, and can be activated under a range of stress conditions, in addition to being engaged by secondary messengers including Ca^2+^ and ROS (Jalmi and Sinha, 2015). Such MAPK signaling is closely associated with responses to pathogens (Gong et al., 2019), temperature stress, heavy metals, wounding (Zhou et al., 2009), and drought (Smekalova et al., 2014). Few studies have specifically explored the role of MAPK signaling in the context of nutrient stress, suggesting that this is a valuable avenue for future research. Herein, we found miR397 to be upregulated under low-K^+^ conditions. This miRNA has previously been linked to the regulation of plant tolerance to salinity and drought stress *via* the regulation of laccase expression (Cho et al., 2014; Gupta et al., 2014). In order to ensure normal enzymatic functionality, it is essential that plants maintain a high cytosolic K^+^-to-Na^+^ ratio (Shabala and Cuin, 2008). Patel et al. (2019) determined that miR397 is upregulated in banana leaves and roots under copper deficiency conditions and is downregulated following treatment with NaCl. Copper ions serve as important enzyme cofactors (Ravet and Pilon, 2013), and the overexpression of this miRNA in bananas failed to interfere with Cu deficiency or NaCl stress tolerance (Patel et al., 2019). Furthermore, miR156 (Liu et al., 2008; Jerome et al., 2020) and miR171 (Hwang et al., 2011), which have previously been linked to plant abiotic stress responses, were also differentially expressed in response to low-K^+^ treatment (Supplementary Table 6). This suggests that many different miRNAs may play important roles in controlling growth and tolerance for different biotic stressors.

Na^+^/K^+^ homeostasis in the cytosol is crucial for plant growth (Almeida et al., 2017). High salinity always leads to the K^+^ deficiency in plants (Nieves-Cordones et al., 2014; Zhang et al., 2019), and such K^+^ deficiency also increases the deleterious effects of salt stress (Hafsi et al., 2017). In this study, we found that there are overlapping points in plant responses to low-K^+^ and salt stress. Much like DEMs in sugarcane associated with low-K^+^ conditions, targets of DEMs under salt stress were predicted to be involved in catalytic activity (Figure 5; De Paola et al., 2012). MAPK signaling was reported to be involved in plant response to salt stress. Expression of *AtMPK1*, *AtMPK2*, and *AtMKK3* was induced by salt stress, and the overexpression of *AtMKK3* can enhance the salinity tolerance of Arabidopsis (Hwa and Yang, 2008). The KEGG pathway analysis in this study revealed that 14 targets of DEMs were enriched in MAPK signal transduction pathways (Supplementary Table 12). In conclusion, plant genes are involved in MAPK signal transduction pathways, and responses to oxidative stress may be co-regulated under both conditions of K^+^ deficiency and Na^+^ application. The miR156 family is conserved in plants (Aung et al., 2015) and plays critical roles in various biological processes, including developmental regulation and responses to stressors (Jerome et al., 2020). Overexpression of miR156 results in a delay of phase transition and flowering (Fu et al., 2012; Shikata et al., 2012), as well as an increase in biomass (Gao et al., 2016). Expression of miR156 can be induced by drought stress (Bertolini et al., 2013), and overexpression of miR156 was found to enhance plant tolerance to salt and drought stress (Cui et al., 2014). In this study, we found that the expression of miR156-z/x in sugarcane was induced by low-K^+^ stress. miR156 was reported to be involved in plant root development and branching (Xie et al., 2012; Yu et al., 2015). Plants overexpressing miR156 also exhibited more lateral roots, with this likely being attributable to increased K^+^ uptake by plants as a consequence of the upregulation of miR156 and an increase in lateral roots under K^+^ deficient conditions.

MiR171 is also involved in plant responses to low-K^+^ stress. Expression of miR171 in wheat (*Triticum aestivum* L.) was significantly upregulated by low-K^+^ treatment for 4 days (Thornburg et al., 2020). After a 7-day low-K^+^ treatment period, the expression of miR171 was lower in low-K^+^ tolerant Tibetan wild barley (*Hordeum vulgare* L.) cultivar XZ153 relative to the sensitive ZD9 cultivar. miR171 targets and downregulates the expression of methylthioribose kinase 1 (MTK), which is one of the key enzymes involved in the ethylene (ET) biosynthesis process (Ye Z. et al., 2020). As a crucial phytohormone and signaling molecule in the context of plant responses to low-K^+^ stress, ET production must be appropriately regulated (Jung et al., 2009). Results in this study revealed that the expression of miR171 in sugarcane root was initially decreased by the low-K^+^ treatment, after which it increased from 48 to 72 h. Overall, we speculate that the downregulation of miR171 during the early stages of low-K^+^ treatment may facilitate signaling through the ET pathway such that upregulating miR171 is essential for maintaining the normal growth of plants under low-K^+^ conditions.

Owing to the lack of a high-quality reference genome, the Pacbio full-length transcriptome results were used as a reference in this study. However, missing information pertaining to intergenic regions hampered the underlying data analyses, particularly with respect to novel miRNA identification and target prediction. In addition, all of our results were obtained based on transcriptomic data and remain to be experimentally verified. Overall, more research must be done to investigate the regulatory mechanisms whereby miRNAs control plant responses to low-K^+^ stress.

miRNAs involved in sugarcane responses to low-K^+^ stress were identified through a comprehensive analysis of patterns of miRNA expression in sugarcane roots exposed to low-K^+^ stress conditions. We found that both miR156-z/x and miR171-x may play crucial roles in these responses by, respectively, inducing more lateral root formation and by regulating ethylene synthesis. GO and KEGG-based annotation and functional enrichment analysis suggested that the predicted miRNA targets participate in many pathways. Notably, both MAPK signaling and plant hormone signal transduction serve as critical regulators of plant low-K^+^ stress responses. Future analyses, however, will be necessary to confirm and expand upon these findings. The miRNAs identified herein and associated functional analyses highlight a new direction for the investigation of plant responses to low-K^+^ stress and the breeding of low-K^+^ tolerant crops.

## Data Availability Statement

The original contributions presented in the study are publicly available. This data can be found here: National Center for Biotechnology Information (NCBI) BioProject database under accession number .

## Author Contributions

NZ, QZ, and YQ: conceptualization. XF: methodology. ZW: software. NZ, YH, and HL: validation. NZ and XG: data curation. XF, NZ, and XG: writing. NZ, JW, and YQ: project administration and funding acquisition. All authors have read and agreed to the published version of the manuscript.

## Conflict of Interest

The authors declare that the research was conducted in the absence of any commercial or financial relationships that could be construed as a potential conflict of interest.

## Publisher’s Note

All claims expressed in this article are solely those of the authors and do not necessarily represent those of their affiliated organizations, or those of the publisher, the editors and the reviewers. Any product that may be evaluated in this article, or claim that may be made by its manufacturer, is not guaranteed or endorsed by the publisher.

## Footnotes

1
https://www.ncbi.nlm.nih.gov/

2
https://www.sanger.ac.uk/tool/rfam/

3
http://bioconductor.org

4
https://www.arabidopsis.org/cgi-bin/patmatch/nph-patmatch.pl

## Supplementary Material

Supplementary Figure 1 Statistics corresponding to the 5′ terminal nucleotides of miRNAs; G: 5′-guanine, A: 5′-adenine, T: 5′-thymine, C: 5′-cytosine.

Supplementary Figure 2 Expression trend profiles (*p*-value > 0.05) corresponding to several DEMs in sugarcane roots after low-K^+^ treatment. The *x*-axis represents time after low-K^+^ treatment from 0 to 72 h. The y-axis represents the log2 fold change in miRNA expression.

Supplementary Table 1 Statistics corresponding to small RNA sequencing data from all 18 libraries generated from sugarcane root samples.

Supplementary Table 2 Details regarding known miRNAs identified in sugarcane roots.

Supplementary Table 3 Details regarding novel miRNAs identified in sugarcane roots.

Supplementary Table 4 The distribution of known miRNAs in the 6 sugarcane root samples.

Supplementary Table 5 The distribution of novel miRNAs in the 6 sugarcane root samples.

Supplementary Table 6 Statistics pertaining to identified miRNA 5′-end nucleotides.

Supplementary Table 7 Differentially expressed miRNAs (DEMs) in sugarcane root samples after low-K^+^ treatment.

Supplementary Table 8 Expression trend profiles of DEMs in sugarcane roots after low-K^+^ treatment.

Supplementary Table 9 Target mRNAs of all the identified miRNAs.

Supplementary Table 10 Target mRNAs of differentially expressed miRNAs (DEMs).

Supplementary Table 11 GO annotation of putative target mRNAs of DEMs.

Supplementary Table 12 KEGG annotation of putative target mRNAs of DEMs.

Supplementary Table 13 Primers for qPCR or stem-loop RT-qPCR used in this study.
