# Supplementary figures and images for: Integrated Analysis of miRNAs Associated With Sugarcane Responses to Low-Potassium Stress
Source: Front Plant Sci. 2022 Jan 4;12:750805. doi: 10.3389/fpls.2021.750805 (PMC8763679; doi:10.3389/fpls.2021.750805)

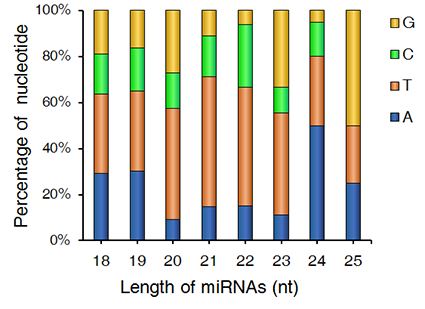

Supplement: Supplementary Figure 1 — Statistics corresponding to the 5′ terminal nucleotides of miRNAs; G: 5′-guanine, A: 5′-adenine, T: 5′-thymine, C: 5′-cytosine. [file Image_1.tif]

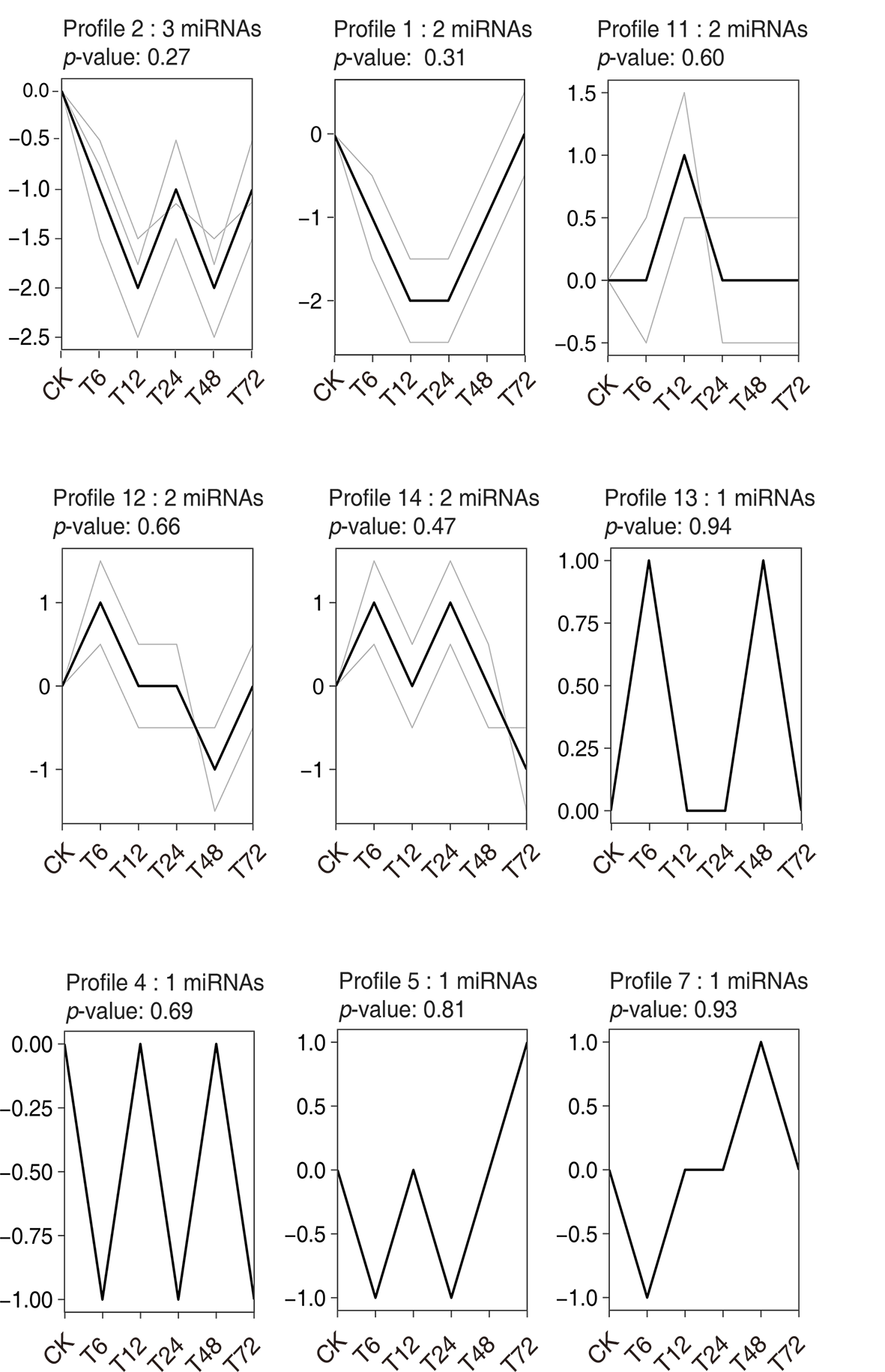

Supplement: Supplementary Figure 2 — Expression trend profiles (p-value > 0.05) corresponding to several DEMs in sugarcane roots after low-K+ treatment. The x-axis represents time after low-K+ treatment from 0 to 72 h. The y-axis represents the log2 fold change in miRNA expression. [file Image_2.tif]
